# Extracellular RNAs released by plant-associated fungi: from fundamental mechanisms to biotechnological applications

**DOI:** 10.1007/s00253-023-12718-7

**Published:** 2023-08-12

**Authors:** An-Po Cheng, Seomun Kwon, Trusha Adeshara, Vera Göhre, Michael Feldbrügge, Arne Weiberg

**Affiliations:** 1 Faculty of Biology, Ludwig-Maximilians Universität München (LMU), 82152 Martinsried, Germany; 2 Institute for Microbiology, Heinrich Heine Universität Düsseldorf, 40225 Düsseldorf, Germany

**Keywords:** Extracellular RNAs, Cross-kingdom RNA interference, Extracellular vesicles, RNA spray

## Abstract

**Abstract:**

Extracellular RNAs are an emerging research topic in fungal-plant interactions. Fungal plant pathogens and symbionts release small RNAs that enter host cells to manipulate plant physiology and immunity. This communication via extracellular RNAs between fungi and plants is bidirectional. On the one hand, plants release RNAs encapsulated inside extracellular vesicles as a defense response as well as for intercellular and inter-organismal communication. On the other hand, recent reports suggest that also full-length mRNAs are transported within fungal EVs into plants, and these fungal mRNAs might get translated inside host cells. In this review article, we summarize the current views and fundamental concepts of extracellular RNAs released by plant-associated fungi, and we discuss new strategies to apply extracellular RNAs in crop protection against fungal pathogens.

**Key points:**

• *Extracellular RNAs are an emerging topic in plant-fungal communication.*

• *Fungi utilize RNAs to manipulate host plants for colonization.*

• *Extracellular RNAs can be engineered to protect plants against fungal pathogens.*

## Introduction

Fungal-plant interactions can have beneficial, detrimental, or neutral effects on plant hosts. Pathogenic fungi pose serious threats to agronomic yield and ecosystems (Fisher et al. 2020), and innovative strategies for controlling these notorious pathogens are needed. Decades of research have been spent to unravel the function of fungal extracellular proteins, effectors, and toxins and their contribution to fungal pathogenesis and disease (Giraldo and Valent 2013; Lo Presti et al. 2015).

Small RNAs are known players in the gene regulatory mechanism often referred to as RNA interference (RNAi) that is largely conserved between fungi and plants. Key factors of RNAi, namely RNA-dependent RNA polymerase (RDR), Dicer-like (DCL), and Argonaute (AGO) proteins, are highly conserved in both plants and fungi (Bologna and Voinnet 2014; Chang et al. 2012). RDR-produced double-stranded (ds)RNAs are cleaved by type-III RNA endonucleases DCL, resulting in mature small interfering RNA (siRNA) duplexes of 21–25 nucleotides in length. DCLs also produce microRNA (miRNA) from hairpin-structured RNA precursors in an RDR-independent fashion. The guide strand of siRNA/miRNA duplexes is loaded onto AGO proteins to form the RNA-induced silencing complex (RISC). This complex can silence RNAs with sequences complementary to small RNAs at the transcriptional or post-transcriptional level. The latter occurs through cleavage of target mRNAs by the AGO endonuclease activity. Shared functions of RNAi in fungi and plants are antiviral immunity, transposon, and transgene silencing, as well as endogenous gene regulation. Among these roles, small RNAs are recognized to impart significant contributions in regulating plant immunity and are proposed to also play crucial roles in fungal pathogenesis (Huang et al. 2019; Qiao et al. 2021b; Weiberg et al. 2014). A fascinating phenomenon is that small RNAs can move between cells and tissues to induce systemic RNAi in plants (Maizel et al. 2020), while extracellular small RNAs produced by fungi mediate cross-kingdom RNAi in plants during host colonization.

In recent years, research on extracellular RNA communication between fungi and plants has emerged as a new topic in plant–microbe interaction (Wang and Dean 2020; Weiberg et al. 2015, 2014). Extracellular small RNAs are secreted by pathogenic as well as beneficial fungi that can enter cells of respective plant hosts to induce cross-kingdom RNAi. Fungal small RNAs silence genes in *trans* within an interacting organism of a different kingdom to promote infection. One potential mechanism of RNA transport from fungi into plants is via extracellular vesicles (EVs). EVs are nanoparticles encasing cytoplasmic molecules including proteins and RNAs in a lipid bilayer, which are secreted into the extracellular space (Colombo et al. 2014). It became evident that cell wall–containing organisms such as bacteria, fungi, and plants release diverse types of EVs. While EV-packaged RNAs have been already associated with plant immunity during fungal and bacterial infections (de la Canal and Pinedo 2018; Rutter and Innes 2018; Rybak and Robatzek 2019), we are only beginning to understand that plant-associated fungi also release EVs, but their function in host infection is not understood. EVs released by animal-associated fungi were reported to play a positive role for pathogenesis (Bielska and May 2019; Zamith-Miranda et al. 2018). Moreover, an increasing number of studies analyzing EVs released by both plant- and animal-associated fungal species led to the detection of not only small RNAs, which are presumed to induce cross-kingdom RNAi, but also full-length mRNAs as cargo, which might get translated in the recipient host cell (Kwon et al. 2021).

Gaining a better understanding of the molecular functions and the roles of fungal extracellular RNAs and EVs in plant infection has a great potential of opening new avenues to invent novel plant protection strategies. While previous reviews have focused on plant-derived extracellular RNAs and EVs in host-microbe interactions (Cai et al. 2021, 2019; Ruf et al. 2022; Stotz et al. 2022), this review highlights the recent discoveries and concepts of extracellular RNAs and EVs released by plant-associated fungi and the potential of utilizing this information to design innovative biotechnological applications for crop protection.

## Fungal small RNAs and cross-kingdom RNA interference

**Fig. 1:**
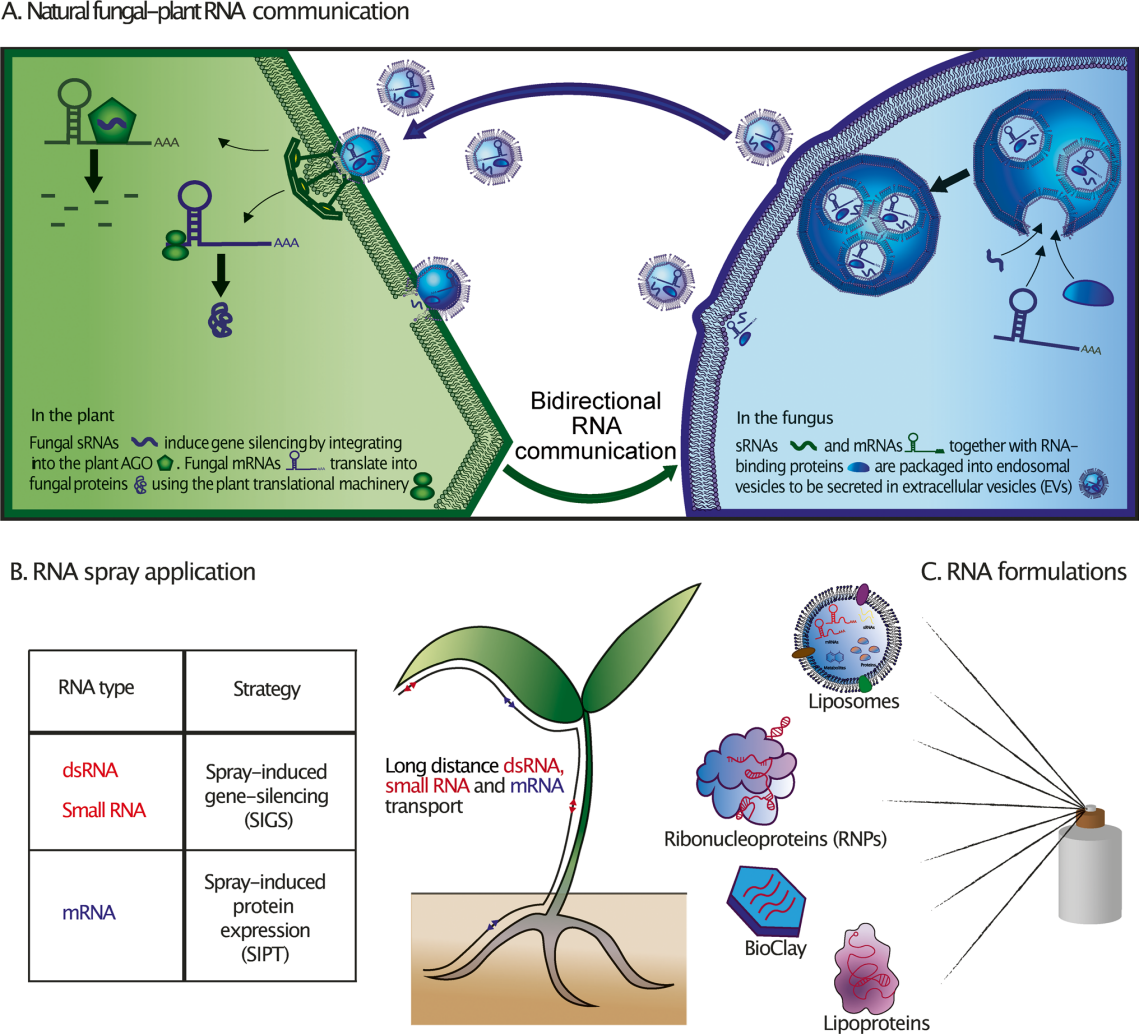
A current model of fungal-plant RNA communication and its implication on RNA spray application. **A** Fungal small RNAs and mRNAs are packaged into extracellular vesicles as potential means of transport into plant host cells. Fungal small RNAs bind to the plant’s own Argonaute/RNA-induced silencing complex to silence plant mRNAs. Fungal mRNAs might load into the plant’s own translational machinery to outsource fungal protein production into plant host cells. RNA communication between fungi and plants is bidirectional, as plants deliver small RNAs and potentially mRNAs into interacting fungi. **B** Current RNA spray applications are based on dsRNA precursors and mature small RNAs for spray-induced gene silencing against fungal pathogens. Future applications may be extended to mRNAs to produce inhibiting peptides inside fungi. **C** RNA formulations have been developed to increase sprayed RNA stability and target delivery

A current model of fungal-plant RNA communication is shown in Fig. 1A. The concept of cross-kingdom RNAi has been established based on host-induced gene silencing (HIGS). Expression of antifungal dsRNAs in barley could induce gene silencing in the powdery mildew pathogen *Blumeria graminis* (Nowara et al. 2010). Since then, the HIGS strategy has been consolidated in diverse fungal-plant interactions, limited to not only in the pathogenic but also in the symbiotic arbuscular mycorrhiza interaction between *Rhizophagus irregularis* and its host plant *Medicago truncatula* (Hartmann et al. 2020).

Another milestone was the discovery of the natural occurrence of cross-kingdom RNAi. *Botrytis cinerea* delivers small RNAs into plant cells that bind to the plant’s own AGO1 to silence host genes that are vital for plant immunity (Weiberg et al. 2013). Five *B. cinerea* small RNAs that induce cross-kingdom RNAi have been functionally characterized so far (Wang et al. 2017; Weiberg et al. 2013). Remarkably, cross-kingdom RNAi is a common natural phenomenon in diverse plant-biotic interactions not only restricted to fungal pathogens (Weiberg et al. 2015) but also exists in oomycetes (Dunker et al. 2020), parasitic plants (Shahid et al. 2018), and fungal as well as bacterial symbionts (Ren et al. 2019; Wong-Bajracharya et al. 2022). These cases of cross-kingdom RNAi were reported in highly diverse biotic interactors of plants comprising different lifestyles and interacting with different host plant species. Typically, these mutualistic and parasitic small RNAs manipulate host gene expression by exploiting the plant AGO proteins, seemingly being an Achilles’ heel that cannot differentiate between self and nonself small RNAs (Dunker et al. 2020; Ji et al. 2021; Ren et al. 2019; Weiberg et al. 2013).

Most of the *B. cinerea* small RNAs inducing cross-kingdom RNAi are derived from retrotransposons that became pathogenicity factors in this fungus (Porquier et al. 2021). Transposons are general hot spots of small RNA production in fungal pathogens (Raman et al. 2013), and their high sequence variation provides an ideal playground to target multiple plant mRNAs in diverse host species. This random gene targeting mechanism by pathogen small RNAs has been proposed as a “shotgun strategy” (Hudzik et al. 2020) that would be beneficial for multitrophic pathogens such as *B. cinerea* to infect diverse plant species.

The fungal vascular pathogen *Fusarium oxysporum* induces cross-kingdom RNAi to achieve tomato root infection. For this, *F. oxysporum* small RNAs bind to the tomato Sl-AGO4a, the ortholog of the *Arabidopsis thaliana* AGO4 (Ji et al. 2021). *A. thaliana* AGO4 mainly associates with 24-nt long heterochromatic siRNAs to silence transposons via RNA-directed DNA methylation (RdDM) (Matzke and Mosher 2014), a unique RNAi pathway existing in plants but not in fungi (Freitag et al. 2004). Whether fungal small RNAs associated with plant AGO4 orthologs can enter the plant nucleus to induce de novo DNA methylation in the host was not examined in the original work. Therefore, both post-transcriptional mRNA cleavage and RdDM are two plausible pathways of target silencing. The oomycete pathogen *Hyaloperonospora arabidopsidis* employs small RNAs that associate with the *A. thaliana* AGO1 to induce cross-kingdom RNAi (Dunker et al. 2020), a striking similarity to *B. cinerea*–induced cross-kingdom RNAi. Oomycetes belong to the phylogenetic group of Chromista, a eukaryotic kingdom that diverged from fungi over 1.5 billion years ago (Parfrey et al. 2011). Moreover, *H. arabidopsidis* is an obligate biotrophic pathogen that is highly adapted to its sole host plant *A. thaliana*. A long co-evolutionary history of the *H. arabidopsidis*–*A. thaliana* relationship is illustrated by an ongoing arm-race (Baxter et al. 2010). One would expect that co-evolution is reflected in cross-kingdom RNAi, in which target gene variation to escape silencing should be followed by pathogen small RNA sequence adaptation. Such co-evolutionary race in trans-species RNAi has been suggested in the parasitic plant genus *Cuscuta* (Johnson et al. 2019). The species *Cuscuta campestris* silences host immunity genes with a subset of 22-nt long miRNAs (Shahid et al. 2018). These miRNAs are conserved in several parasitic *Cuscuta* species, in which they have evolved to generate larger miRNA families comprising compensatory sequence variations according to the binding sites in the host target genes (Johnson et al. 2019). Owing to this compensatory sequence variation, *Cuscuta* might be able to quickly adapt miRNAs to keep functionality in trans-species RNAi by matching with host target allelic variants. The ectomycorrhizal fungus *Pisolithus microcarpus* delivers miRNA-like RNAs (milRNAs) into the root cortex of its host plant *Eucalyptus grandis*. Treatment of roots with synthetic *Pisolithus* milRNAs mimicked regulation of *Eucalyptus* target genes and strengthened formation of deep Hartig net during root colonization (Wong-Bajracharya et al. 2022). Cross-kingdom RNAi is a common strategy among the distinct plant root colonizing microbes, encompassing not only eukaryotic pathogens and symbionts but also prokaryotes. The gram-negative bacterium *Bradyrhizobium japonicum* delivers small RNAs into soybean (*Glycine max*) roots in order to establish root nodule symbiosis (Ren et al. 2019). Bacteria lack a canonical RNAi pathway and do not possess DCL type-III RNA nucleases. Nevertheless, *B. japonicum* delivers transfer RNA-derived small RNAs (tRFs) into the soybean AGO1b to induce cross-kingdom RNAi of nodule-repressive plant genes. Interestingly, both cases of cross-kingdom RNAi help to establish distinct forms of root symbiosis, in which microbial small RNAs seem to act as early stage interaction signals, because RNA delivery into plant cells occurs before the formation of fungal Hartig net and bacterial nodules.

It is important to note that not all fungal plant pathogens rely on extracellular small RNAs, as lack of cross-kingdom RNAi and HIGS was reported in the fungal wheat pathogen *Zymoseptoria tritici* (Kettles et al. 2019; Ma et al. 2019). Also, the model smut fungus *Ustilago maydis* lost DCL and AGO over evolution, which are key components for small RNA biogenesis and RNAi (Laurie et al. 2008); however, *U. maydis* might use DCL-independent small RNAs for cross-kingdom RNA communication.

## Fungal extracellular vesicles as carriers of RNA

A key question in exRNA-mediated communication between fungi and plants is how RNAs are transported between interacting organisms. EVs represent one of the potential mechanisms of exRNA transport. The existence of fungal and plant EVs has been reported over the last two decades, although this has been controversially discussed due to questions of how EVs might traverse via the plasma membrane and cell wall. While the origin and the identity of fungal EVs had been discussed (Coelho and Casadevall 2019; McMillan and Kuehn 2021), suitable protocols for EV isolation and analysis of their molecular cargo are now available to address these points. Regarding the cell wall as a barrier, the fungal cell wall is considered to be a highly dynamic structure with pore sizes up to hundreds of nanometers wide that could allow passage of EVs (Brown et al. 2015; Ebrahimi et al. 2023). Liposomes, which are comparable to natural EVs, can pass through the fungal cell wall due to their viscoelastic properties (Walker et al. 2018). Moreover, cell wall remodeling enzymes have been consistently detected in fungal EV proteomes and may mediate local loosening of the cell wall to allow passage of EVs (Zhao et al. 2019).

Intimate contact sites where fungal hyphae or feeding structures are encased by the host plant plasma membrane are likely spots for EV-mediated RNA exchange. EV-like structures from both plants (An et al. 2006) and plant-colonizing fungi (Ivanov et al. 2019; Ludwig et al. 2021; Roth et al. 2019) have been observed accumulating at such contact sites. The maize smut fungus, *Ustilago maydis*, produces both paramural vesicles contained within the fungal cell wall (Roth et al. 2019), as well as membrane protrusions beyond the fungal cell wall, surrounded by the maize plasma membrane (Ludwig et al. 2021). These fungal membrane protrusions harbor a protein complex, which not only mediates effector delivery but also interacts with various proteins in the maize plasma membrane, including aquaporins. In *A. thaliana*, aquaporins are endocytosed upon salicylic acid–induced ROS stress (Boursiac et al. 2008). Borrowing from the model of bacterial effector translocation via endocytosis with plant aquaporins (Zhang et al. 2019), fungal EVs and RNAs may also target plant aquaporins for uptake. Furthermore, clathrin-mediated endocytosis is a major route of uptake for filamentous pathogen effectors targeted to the plant cytosol (Oliveira-Garcia et al. 2023; Wang et al. 2023), and co-uptake of conventionally secreted fungal effectors and EVs may be possible. Preliminary data support the notion that RNAs loaded into fungal EVs might enter into *A. thaliana* cells via clathrin-mediated endocytosis (He et al. 2023). While endocytosis is emerging as a probable mode of EV and RNA uptake into plant cells, EV cargo release and delivery to the host cytosol would require fusion with the limiting membrane of the endosomes. Factors required for endosomal escape of EV cargos remain to be elucidated.

As mRNAs are recognized as common, bona fide cargos of EVs (O’Brien et al. 2020), it is hypothesized that plants and microbes exchange mRNAs that may be translated into functional proteins in recipient cells. Reads from coding transcripts had long been detected in sequencing of fungal EV-associated small RNAs (Peres da Silva et al. 2015). mRNAs are more recently being analyzed in earnest as fungal EV cargos, although the biological purpose of their secretion remains unclear (Alves et al. 2019; Kwon et al. 2021; Peres da Silva et al. 2019; Zamith-Miranda et al. 2018). While current studies on fungal EV-associated mRNAs are descriptive, they provide a glimpse into potential biological functions and mechanisms of mRNA loading into EVs. Among phytopathogenic fungi, EV-associated mRNAs were first extensively cataloged in the maize smut pathogen, *Ustilago maydis* (Kwon et al. 2021). The presence of intact, spliced, and poly(A)-tailed mRNAs in *U. maydis* EVs was evident, albeit with lower integrity overall, compared to intracellular transcripts, as reported in mammalian systems (Hinger et al. 2018). Comparable to findings in human cell lines (Hinger et al. 2018; O’Grady et al. 2022), shorter mRNAs were relatively enriched in *U. maydis* EVs, with a median ORF length of ~ 1 kb, while longer transcripts were relatively underrepresented in EVs. Given that the EV-associated transcript profiles remained similar regardless of external RNase treatment (Kwon et al. 2021), it is likely that mRNAs are protected within the EV lumen, although EV-independent modes of RNA secretion and delivery cannot be ruled out.

While a vast majority of mRNAs transcribed in *U. maydis* cells could be detected in the heterogeneous EV population, a subset of transcripts was relatively enriched in EVs. For example, transcripts encoding cytosolic metabolic enzymes were particularly overrepresented in EVs; these may bring about amplifiable physiological changes to the plant host when translated in the recipient cells. Furthermore, this reflects the capacity of the smut fungus to reprogram host plant metabolism during infection (Doehlemann et al. 2008). Subcellular localization of the mRNAs may also influence their loading into EVs. mRNAs are often transported and locally translated where the protein products are required, as previously reviewed (Das et al. 2021; Muntjes et al. 2021). Based on the data from *U. maydis* EVs, mRNAs encoding endosomal or cytosolic proteins were more likely to be overrepresented in EVs than those that must be targeted to the ER (Kwon et al. 2021). Thus, proximity of an mRNA to limiting membranes of maturing endosomes or the cell periphery could increase their chances of being incorporated into exosomes or microvesicles, respectively.

The process of selective RNA loading and secretion via EVs is not understood in detail, but as is common for intracellular RNA transport, entails RNA-binding proteins (RBPs) and mRNAs with cognate motifs for targeting them to sites of EV biogenesis. In mammalian systems, multiple RBPs have been implicated in RNA loading into EVs (Fabbiano et al. 2020). For example, in EVs from human umbilical vein endothelial cells, enriched mRNAs harbor structural features linked to increased stability, as well as motifs for HNRNPA2B1-binding (O’Grady et al. 2022). Interestingly, retrotransposon-derived ARC proteins, convergently co-opted in human and fruit fly, form virus-like capsids, bind their own mRNA, and are secreted from neurons via EVs (Ashley et al. 2018; Pastuzyn et al. 2018). Such virus-like mechanisms of exRNA transport await discovery in plants and fungi. An extensive RBP correlation footprinting analysis based on eCLIP data of 150 human RBPs with exRNA reads has found sequences from at least 30% of all human protein-coding genes (LaPlante et al. 2023). Moreover, mRNA-derived sequences were significantly enriched with EV-associated RBPs compared to other EV-independent RBPs, supporting that EVs are the major means of mRNA secretion. The presence of various canonical and non-canonical mRNA-binding proteins in EV proteomes of mammalian cell lines further supports the role of RBPs in mRNA loading into EVs (Castello et al. 2012; Pathan et al. 2019). In *A. thaliana*, selective sorting of small RNAs that induce cross-kingdom RNAi into EVs is facilitated by AGO1 and the two DEAD-box RNA helicases (RH)11 and RH37, which all specifically bind to the EV small RNAs, as well as the two non-specific RBPs annexins (ANN)1 and ANN2 (He et al. 2021). It is probable that orthologs of these proteins may be responsible for selective RNA loading into fungal EVs. Given that annexins are mRNA-binding proteins in mammalian cells (Strand et al. 2021), they might also mediate mRNA cargo selection into fungal EVs.

EVs may be a mechanism for delivering proteins lacking signal peptides for conventional secretion, in the form of either protein or mRNA to be translated *in planta*. Median translation rate, estimated in mammalian cells, can be over 100 protein molecules per mRNA per hour (Schwanhausser et al. 2011), and a single mRNA can yield from a few hundred to hundreds of thousands of protein molecules (Edfors et al. 2016). If fungal mRNAs are translated into effector proteins in host plant cells, which in turn produce amplifiable physiological effects, it could be a highly cost-effective strategy for the pathogen. In the clinically important fungus *Paracoccidioides brasiliensis*, the presence of intact, translation-competent, EV-associated mRNAs was demonstrated by in vitro translation of the extracted RNA, followed by proteomic analysis (Peres da Silva et al. 2019). While this approach has led to detection of only a handful of proteins, it was a proof of concept that the EV-associated mRNAs can be translated using a heterologous system. It remains to be determined whether compatibility of factors such as codon usage preference, untranslated regions, and RBPs would allow sufficient translation efficiency to yield a physiologically relevant level of fungal protein in the host. Delivery of pathogen mRNAs and their translation in host plant cells still must be demonstrated, and a clear biological function has yet to be attributed to candidate mRNA effectors. Nonetheless, effector delivery in the form of mRNAs is a fascinating and theoretically probable hypothesis (Kwon et al. 2021).

## RNA communication from plants to fungi

Extracellular RNAs and EVs are produced by both fungi and plants. Effective silencing of fungal genes by HIGS emphasizes that cross-kingdom RNAi is bidirectional in fungal-plant interactions (Wang et al. 2016). As a natural defense mechanism, cotton plants transfer miRNAs into the vascular pathogen *Verticillium dahliae* that cleave *V. dahliae* mRNA targets (Zhang et al. 2016). The cotton miRNAs were detectable in the mycelium up to 20 days post re-isolation from infected cotton tissue, indicating a potential amplification loop of exogenous plant small RNAs after intruding into the fungal cells. The relevance of fungal RNAi components, such as RDRs, DCLs, and AGOs, in plant-induced cross-kingdom RNAi still needs to be examined. *A. thaliana* delivers miRNAs and trans-acting (ta)siRNAs into infecting *B. cinerea*. These *A. thaliana* small RNAs are suggested to be transported via plant EVs (Cai et al. 2018), together with the plant AGO1 and two RNA helicases (He et al. 2021), suggesting that RBPs are important factors in small RNA secretion, extracellular RNA stability, and function. Furthermore, enrichment of N^6^-methyladenine (m^6^A) RNAs was found in the plant extracellular fraction (Karimi et al. 2022), which hints to RNA modification as another mechanism to direct RNA secretion and extracellular stability. It is worth to mention that m^6^A RNA profiles were recorded on exRNAs of non-infected plants, while EV-encapsulated exRNAs might be predominantly released upon infectious stress. The discovery of full-length mRNAs in *U. maydis* EVs, as well as bidirectional exchange of mRNAs between *A. thaliana* and the parasitic plant species *C. campestris* (David-Schwartz et al. 2008), suggests plausible bidirectional transfer of mRNAs between fungi and plants, too.

## Applying extracellular RNAs for crop protection

To date, agronomic control of fungal pathogens strongly relies on the application of chemical pesticides. Besides their crop protective effects, some pesticides have harmful side effects on human health, pollute the environment, and force selection for pesticide-resistant pathogen variants (Pathak et al. 2022). New RNA-based pesticide strategies, aka RNA spray, has been developed over the last years (Fig. 1B) that promise to overcome these obstacles.

Since the discovery of cross-kingdom RNAi and its technological implementation into HIGS application, RNAs have been engineered to confer resistance in plants against diverse pathogenic organisms with significant success (Hou and Ma 2019; Koch and Wassenegger 2021; Nunes and Dean 2012). Nevertheless, HIGS is a transgenic approach, which still faces hurdles to gain broader societal acceptance and approval for large-scale application. As a non-GMO approach, spray-induced gene silencing (SIGS) has now been tested in several plant pathology laboratories (Koch et al. 2019). Like the HIGS approach, a dsRNA is directed against an essential gene of a pathogen or pest. A first market-ready product called Calantha™ with the active RNAi compound “ledprona” has been released by the GreenLight Biosciences company, which protects potato plants against the Colorado potato beetle (*Leptinotarsa decemlineata*). Accordingly, essential field trials in the USA are proceeding to pave the way for final approval. Such development of successful SIGS application keeps high hopes that RNA spray also becomes conceivable for plant protection against fungal pathogens in the near future.

In order to develop SIGS-based fungicides, at least three goals need to be conceived. First, a suitable fungal target gene needs to be identified that is effectively downregulated by the RNAi spray and stop pathogen infection. First candidate genes have been approved, such as the fungal *CYP51*s (essential for ergosterol biosynthesis) and *DCL*s (RNAi pathway) (Koch et al. 2016; Wang et al. 2016), which were before successfully targeted by HIGS to confer plant resistance. However, suppressing conserved fungal genes by SIGS may co-inhibit related fungal species comprising target sequence overlaps, too, which may have impacts on the natural fungal microbiome of plants. A strategy to exclusively target genes in pathogenic species could be a next logical step. These genes could be identified in large-scale genome comparisons utilizing the rapidly growing numbers of high-quality genome sequencing data becoming available.

Second, sprayed RNA onto plants must be sustained active against a fungal pathogen over a period of time. Application of “naked” RNA onto leaf and fruit surfaces was capable to suppress fungal infection for few days under controlled condition. In this context, it is still not clear if RNA molecules take a path through the plant tissue, vasculature, or even plant cells before being taken up by the infecting fungus. Using fluorescently labeled RNA molecules, circulation of fluorescence was observed in the plant vasculature (Koch et al. 2016). Moreover, first experiments supported the idea of long-distance RNA transport that could provide systemic protection against fungi. There are also a couple of concomitant challenges for SIGS to achieve lab-to-field transition that has been previously reviewed in detail (Rank and Koch 2021). These challenges are related to RNA formulation and application which includes aspects of RNA stability in the field, methods, and timing of RNA application and profitable costs.

Third, sprayed RNAs should be effectively delivered into target fungi. In a screening of naked RNA application, it turned out that RNA uptake efficiency varies among fungal plant-pathogenic species. While in the cases of *B. cinerea*, *Sclerotinia sclerotiorum*, *Rhizoctonia solani*, *Aspergillus niger*, and *V. dahliae* RNA was readily taken up, *Colletotrichum gloeosporioides* and *Trichoderma virens* exhibited poor RNA uptake efficiencies (Qiao et al. 2021a). Ultimately, RNA uptake as well as HIGS completely failed in the case of *Zymoseptoria tritici* (Kettles et al. 2019). These observations indicate that a potential RNA-based fungicide application needs to be always carefully evaluated. The RNA uptake mechanisms into fungal cells are not understood (Schlemmer et al. 2022), but small RNA transport from plants into fungi is mediated by EVs and EV-associated RBPs (Cai et al. 2018; He et al. 2021), which both might enhance efficiency of RNA uptake into fungal cells. Using such information of naturally occurring cross-kingdom RNAi in plant-fungal interactions seems to be valuable to indicate the suitability for an RNA fungicide application, as demonstrated for the species *B. cinerea* and *V. dahliae* that induce natural cross-kingdom RNAi and are sensitive the RNA spray and *Z. tritici* that does not induce cross-kingdom RNAi and does not take up RNA. With the discovery of full-length protein-coding mRNAs transported via EVs (Kwon et al. 2021), a potential application of mRNA spray for plant protection can be envisioned. Delivery of mRNAs that encode suitable inhibitors or toxins effective against fungal pathogens could expand the RNA portfolio for crop protection, which could be effective in fungi that have lost the capacity for RNAi, such as *U. maydis*.

Since extracellular RNA stability and delivery have been identified as the major challenges to bring SIGS into a success story against fungal pathogens (Hernandez-Soto and Chacon-Cerdas 2021; Rank and Koch 2021), nowadays, a lot of attention is paid on RNA formulations. These are mostly derived from biomedical RNA vaccine or therapeutic strategies and are currently tested in the plant context (Fig. 1C). In this regard, packaging layered clay nanoparticles, called BioClay™, can promote RNA stability for SIGS application. These RNA nanoparticles have been proven to be effective against the different developmental stages of the whitefly (*Bemisia tabaci*) on cotton (Jain et al. 2022) as well as against fungal *B. cinerea* infection in tomato and chickpea under controlled conditions (Nino-Sanchez et al. 2022). Recent discoveries on small RNA and mRNA exchange via EVs in fungal-plant interactions (Goehre and Weiberg 2023; Ruf et al. 2022) have inspired plant biotechnologists to explore liposome-based RNA applications. Indeed, artificial nanovesicles derived from cationic lipid formulations protected sprayed RNAs from rapid degradation and could prolong SIGS durability to protect plant surfaces from *B. cinerea* infection (Qiao et al. 2023). In addition to RNA nanocarriers, coupling RNAs to proteins to form a ribonucleoprotein complex (RNP) and RNA-lipid formulations is expected to further improve stability and delivery efficiencies of RNA molecules. RBPs such as AGOs, RNA helicases, and Annexins, which have been found to bind to extracellular RNAs (He et al. 2021), are promising candidates to form RNPs for improving SIGS application.

The SIGS approach stands for a more eco-friendly plant protection strategy that is already in transition into potential field application in first trails (Rank and Koch 2021; Schlemmer et al. 2022). RNA-based insect control currently spearheads the field. In the future, a range of SIGS-based products are expectable to control microbial pathogens of agronomic important crops, too. Extracellular RNA application is an emerging field not only in plant research but also in biomedicine. RNA therapeutics and vaccines are current and future strategies to combat infections and cure diseases. Before applying these innovative RNA solutions in agriculture, they need to meet safety regulatory requirements and, most importantly, broad societal acceptance (Fletcher et al. 2020; Taning et al. 2021). Since mRNA vaccines have now been widely accepted in biomedicine throughout the COVID-19 crisis, RNA-based plant protection strategies might benefit from this wind of change.
